# The role of skin hydration, skin deformability, and age in tactile friction and perception of materials

**DOI:** 10.1038/s41598-025-95052-4

**Published:** 2025-03-22

**Authors:** Victor H. P. Infante, Maja Fehlberg, Sairam Saikumar, Knut Drewing, Martina C. Meinke, Roland Bennewitz

**Affiliations:** 1 INM - Leibniz Institute for New Materials, Saarbrücken, Germany; 2 Corporate Member of Freie Universität Berlin and Humboldt Universität zu Berlin, Department of Dermatology, Venereology and Allergology, Center of Experimental and Applied Cutaneous Physiology, Charité – Universitätsmedizin Berlin, Berlin, Germany; 3 Department of Physics, Saarland University, Saarbrücken, Germany; 4 Department of Psychology, Justus Liebig University, Giessen, Germany

**Keywords:** Skin physiology, Friction, Perception, Meissner corpuscles, Materials, Physiology, Psychology, Materials science

## Abstract

Friction between fingertip and surface is a key contribution to tactile perception during active exploration of materials. We explore the role of skin factors such as *stratum corneum* thickness and hydration, deformability, elasticity, or density of sweat glands and of Meissner corpuscles in friction and tactile perception. The skin parameters were determined non-invasively for the glabrous skin at the index finger pad of 60 participants. Sets of randomly rough plastic surfaces and of micro-structured fibrillar rubber surfaces were explored as model materials with well-defined parameterized textures. Friction varies greatly between participants, and this variation can be explained to 70% by skin factors for the randomly rough plastic surfaces. The predictability of friction by skin factors is much lower for micro-structured rubber surfaces with bendable fibrils, where 50% of variance is explained for the stiffest fibrils but only 20% for the most bendable fibrils. The participants’ age is the key predictor for their tactile sensitivity to perceive the fibrils, where age is negatively correlated to the density of Meissner corpuscles. The results suggest that *stratum corneum* hydration, skin deformability, and age are important factors for friction and perception in active tactile exploration of materials.

**Supplementary Information:**

The online version contains supplementary material available at 10.1038/s41598-025-95052-4.

## Introduction

The perception of materials by touch depends on material properties and surface structure, but also on tactile exploration strategies and on properties of the skin and the nervous system. We are interested how, and how far basic individual differences in mechanical and physiological skin properties influence the process of tactile exploration and perception. From previous studies we know about perceptual consistencies despite individual differences. Individuals consistently rank sample properties such as softness, roughness, slipperiness^1–4^ and even pleasantness^5–7^ based on touch perception.

Our approach is to determine a broad range of peripheral skin parameters that are available to be measured non-invasively, study a broad age range of healthy participants where we expect considerable variance, and test the influence on a set of measures that are representative for tactile exploration and perception.

One important skin parameter is its capacity to deform under mechanical stress^8^. The importance of skin mechanical properties lies in the comprehension of how the contact with a sample can activate mechanoreceptors during tactile exploration, triggering sensory information^9^. Properties and morphology have been studied extensively for hairy skin and correlations with other parameters such as skin hydration or age have been reported^10–13^. Much less is known about physiological parameters for the glabrous skin of the finger pad which is in contact with materials during tactile exploration.

Tactile perception by the fingertip involves the unique innervation of glabrous skin. Meissner corpuscles are located in the dermal-epidermal junction, closer to the skin surface than Pacinian corpuscles and Ruffini organs^14^. Their density can be determined non-invasively by scanning laser microscopy, a method also applied in this study^15,16^. Meissner corpuscles are relevant for touch perception through their fast-adapting neural response to mechanical stimulation^17,18^. The relations between Meissner corpuscle physiology, touch, perception, and ageing are subject of ongoing research^19,20^.

Friction, the force which resists sliding of the finger over a surface during tactile exploration, plays a key role in tactile perception. The shear deformation in the skin caused by friction activates mechanoreceptors^21,22^, as recently exemplified for the role of contact formation in the perception of friction^23^. Our measures of relevance for tactile exploration and perception are therefore friction as a central characteristic of tactile exploration, basic spatial discrimination, and texture perception as one of the core dimensions of discriminative haptic perception^24^.

The stimuli materials which we chose for the study support our approach by offering one hard and one elastic material with systematically varied micro-structures on their surfaces. Friction, texture perception, and spatial discrimination are then expected to depend on parametrized contact mechanics, while the use of unfamiliar materials and structures minimizes the influence of prior knowledge in participants.

## Results

The results of our study will be presented in four sections. We start with the analysis of skin images and the distribution of skin physiological parameters among the participants. In the second section we summarize all results of friction measurements and the outcome of the perceptional tasks. We then apply one principal component analysis to the physiological parameters and one to friction and perception results to identify common factors. Finally, we will list correlations which indicate dependencies between physiology and friction or perception.

**Fig. 1:**
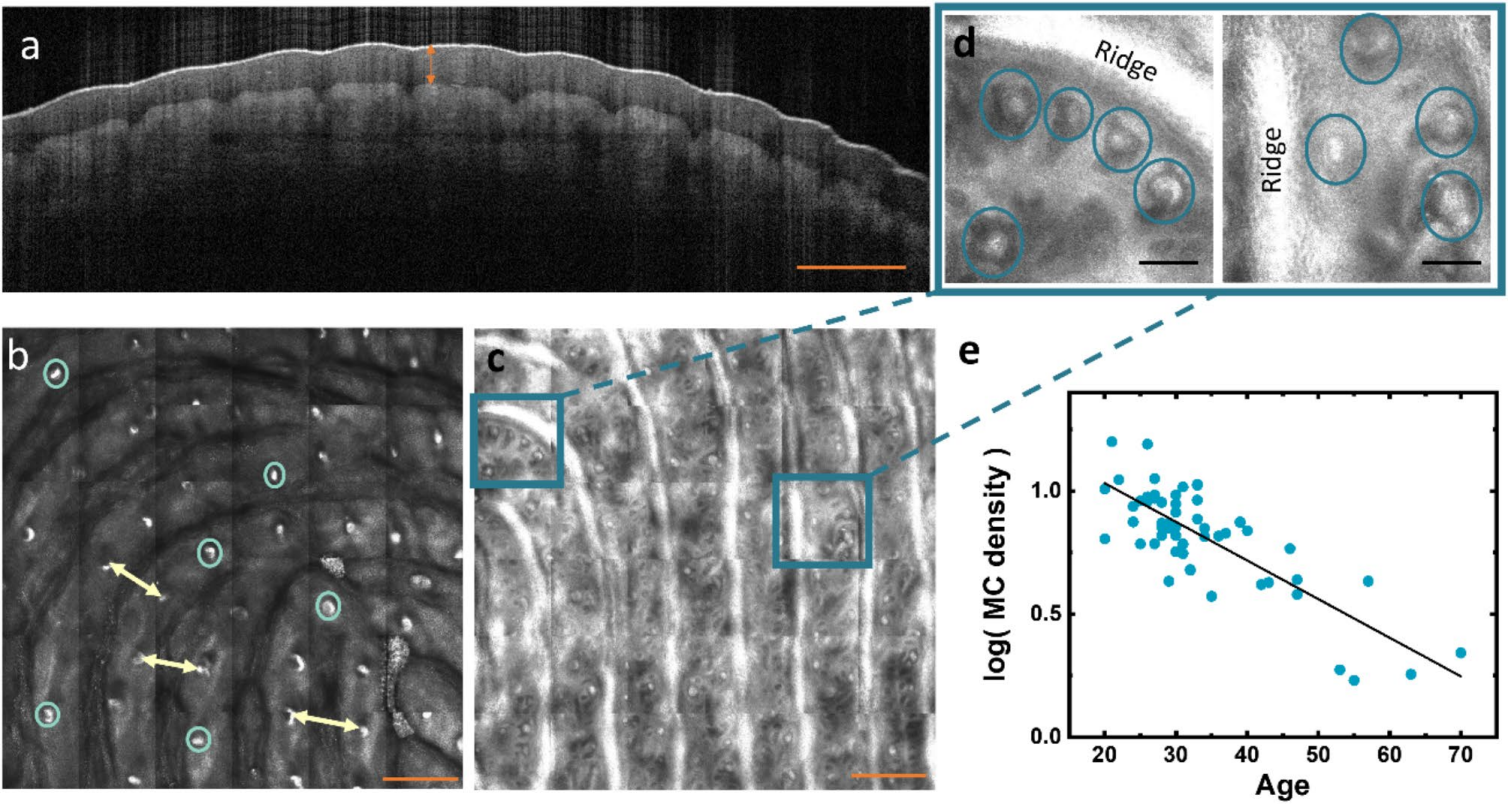
Optical microscopy of skin structure. (**a**) Optical coherence tomography image with a cross-section of the skin surface reveals the thickness of the *stratum corneum* (*SC thickness*) indicated by an orange arrow. (**b**) Laser scanning microscopy (LSM) image recorded at a depth 50 μm in the *stratum corneum*, where some sweat glands are indicated by blue circles. Arrows mark the distance between skin ridges which is determined as distance between sweat glands in adjacent ridges. (**c**) LSM image of a dermal-epidermal junction where Meissner corpuscles (*MC*s) appear as white dots. (**d**) Detailed LSM images of Meissner corpuscles (blue circles). The orange scale bars indicate 500 μm and the black scale bars 100 μm. (**e**) Correlation between age and the density of Meissner corpuscles for 56 participants. The line reports a linear regression to the logarithm of MC density versus age.

### Skin properties

**Fig. 2:**
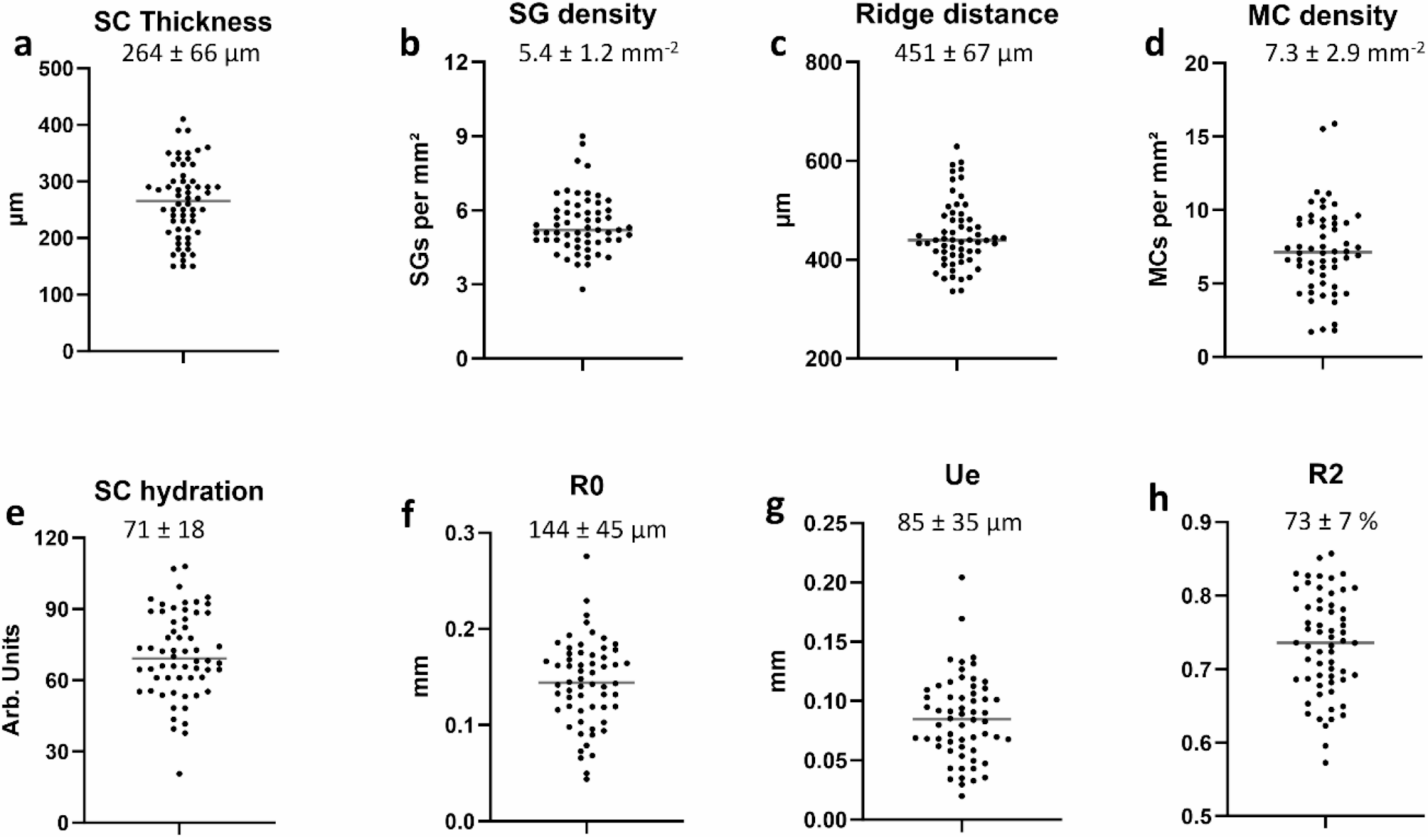
Distribution of skin parameters. (**a**) Thickness of *stratum corneum* (SC thickness) (**b**) Density of sweat glands (SG density) (**c**) Average distance between finger ridges derived from distance of sweat glands (**d**) Density of Meissner corpuscles (MC density) (**e**) Hydration of *stratum corneum* in units of the corneometer (SC hydration) (**f**) Deformability R0 measured by cutometer. (**g**) Fast component Ue (100 ms) of the deformability. (**h**) Elasticity R2 determined as the fraction of deformation which is relaxed after releasing the vacuum suction.

Figure 1 presents three example images which are the basis for determining parameters of the skin structure. Figure 1a is a cross-section of the top skin layers which provides a quantification of the *stratum corneum* thickness (*SC thickness*). The thickness varied considerably between participants, see Fig. 2a–d for distributions of all morphological parameters. Figure 1b shows a plane in the *stratum corneum* (50–75 μm depth) and highlights the sweat glands in the center of each finger ridge. The distance between sweat glands was used to determine the finger ridge distances. Figure 1c shows a dermal-epidermal junction with the characteristic papillary structure and white dots at the sites of Meissner corpuscles (*MCs*). Detailed images clarify the identification of *MCs* by their characteristic appearance close to the edge of papillary ridges in Fig. 1d, for details of the *MC* detection see Ref^25^.

The resolution of LSM images at the depth of the dermal-epidermal junction can be limited by the *SC thickness* on the pads of the index fingers of dominant hands. The density of *MCs* could be determined for only 37 participants from these images. However, we were able to count the *MCs* for 50 participants on the index finger of the non-dominant hand and for 52 participants on the small fingers, where the *SC* is less thick. We found that while the density of *MCs* varies significantly between participants, the density is very similar among the different distal phalanges of one participant. We therefore calculated an average *MC density* for the anatomical positions for all 56 participants from whom we obtained suitable images. The density of *MCs* and its variation between participants agree with earlier studies^19,26^.

Results for the distributions of skin hydration and skin deformability are presented in Fig. 2e–h. We would like to point out that the displacements measured by the cutometer are smaller than the *SC thickness*. The skin deformability *R0* is correlated to the *SC hydration* (*R* = 0.40, *p* < 0.002), but not correlated at all to the *SC thickness*. The skin elasticity *R2* is correlated to both *SC thickness* (*R* = 0.39, *p* < 0.003) and *SC hydration* (*R* = 0.31, *p* < 0.02).

Among the predictor skin parameters, the fast elastic response *U*_*e*_ measured by the cutometer had the largest relative standard deviation of 41%. The density of Meisner corpuscles had a relative standard deviation of 40%. This variation originates in the wide distribution of participants’ age from 20 to 70. A linear regression of the data in Fig. 1e suggests that the density of *MCs* is reduced to one half every 19 years. To estimate the error in this decay time constant, which may arise from an uneven distribution of age across our group of participants, we have performed a bootstrapping analysis of the data set. We find that the 95%-confidence interval for the decay time constant is 15–25 years ($\:-0.65<R<-0.88\:\text{and}\:p<0.001$ for linear fit to all 10^6^ resampled data sets) in agreement with a previous report^26^. The third largest relative standard variation is in the distribution of the skin deformability (R0) (31%), followed by *SC hydration* (25%) and the *SC thickness* (25%). Smaller coefficients of variation are found for the density of sweat glands (21%) and the finger ridge distance (15%). The smallest variation is found for the skin elasticity (*R2*) measured by the cutometer (9%). When we repeat the data analysis for half of the participants in a narrower age distribution (27–36 years, 2nd and 3rd quartile), the relative standard deviations do not change by more than 10% for any parameter except for the density of *MC*s, which is only 26% for this group.

The only skin physiological parameters which exhibited a significant difference between male and female participants was the higher density of sweat glands in females, and the smaller finger ridge distance derived from it (see Table S1 in SI for details). The difference in SG density between the sexes was reported before and has been attributed to a significant difference in finger dimensions, where the SG density was correlated with the finger dimensions^27^. Besides the negative correlation between age and density of Meissner corpuscles, we found a weak negative correlation with age for *SC hydration* (*R* = − 0.31, *p =* 0.017).

**Fig. 3:**
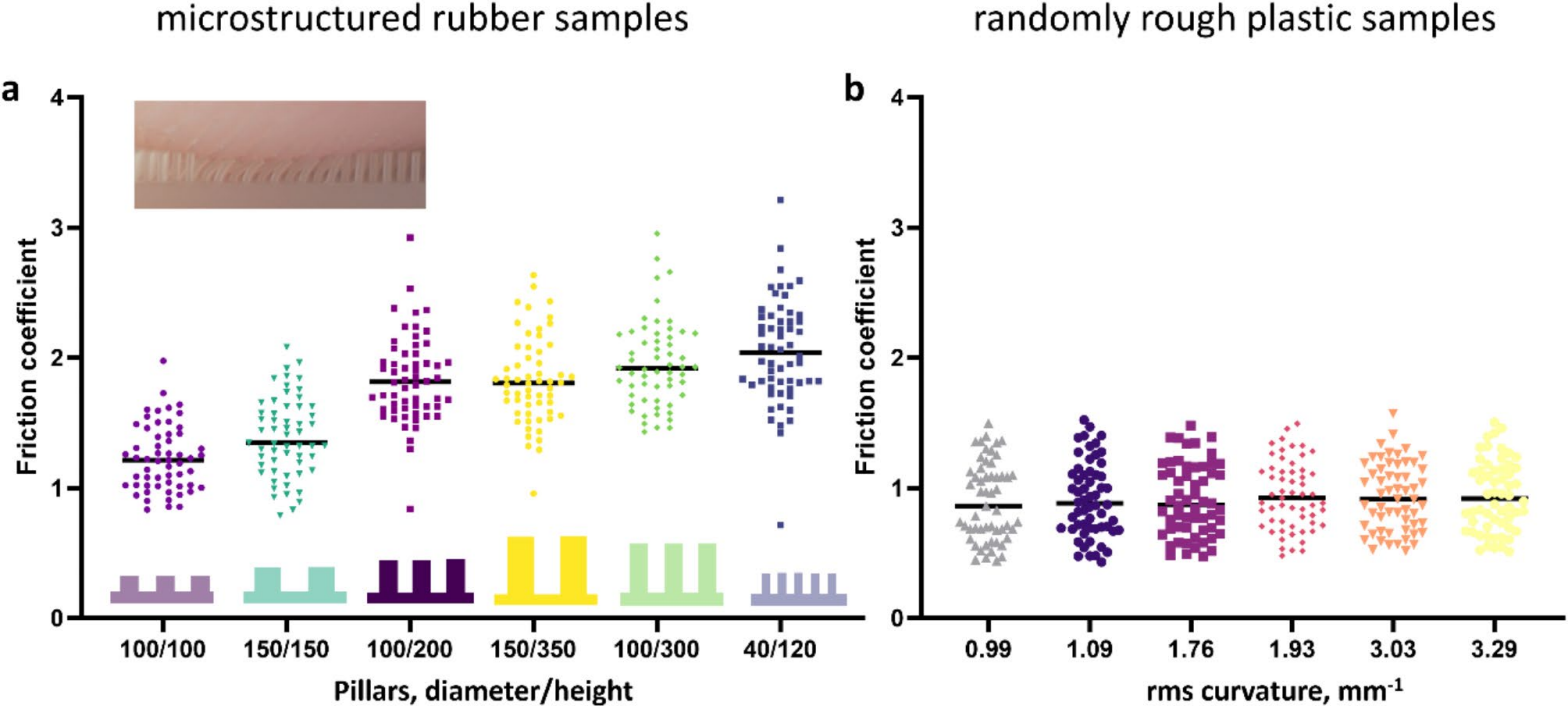
Distribution for the coefficient of friction for the different surfaces. (**a**) Micro-structured rubber samples sorted by aspect ratio of the micropillars. (**b**) Randomly rough plastic samples sorted by increasing values for the RMS curvature, a measure for the roughness at small length scale in units of 1/mm. (**c**) Coefficient of friction averaged over all participants for each randomly rough surface as function of the rms curvature of the surfaces.

### Friction and perception results

We will now present results for the friction measurements. For six micro-structured rubber samples (Fig. 3a), where each surface carries a hexagonal lattice of bendable micropillars, the friction coefficients increased significantly with increasing aspect ratio between height and diameter of micropillars (*R* = 0.64, *p* < 0.001). The friction coefficients ranged from an average of 1.2 for the 100/100 sample to 2.0 for the 40/120 sample, the values indicate diameter/height in µm. The relative standard deviation in the friction coefficient, which can be seen as a measure for individual differences, varied between 17 and 22% for the six samples. The friction coefficients of all participants are positively correlated between samples, with correlation coefficients ranging between *R* = 0.78 (*p* < 0.001) for samples 100/300 and 40/120 to *R* = 0.38 (*p* = 0.004) for samples 100/100 and 40/120.

For six randomly rough plastic samples, the average friction coefficient was 0.92. Friction coefficients did not exhibit significant differences between samples with different small-scale roughness (Fig. 3b). The relative standard deviation in friction coefficients for each sample varied between 28 and 33%. The friction coefficients of all participants are positively correlated between samples with correlation coefficients *R* ≥ 0.94 (*p* < 0.001) for all sample pairs.

Friction is higher by up to a factor of two on the micro-structured rubber samples as compared to the randomly rough plastic samples (Fig. 3). For the micro-structured rubber samples with the lowest friction (100/100 and 150/150) the coefficients were still 1.2–1.5 times higher than for the randomly rough plastic samples.

**Fig. 4:**
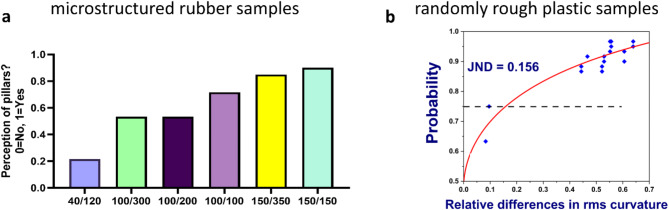
Results of perceptional tasks. (**a**) Probability to perceive the presence of pillars on micro-structured rubber sample in sliding touch. (**b**) Probability for indicating the sample as rougher for which rms curvature was higher versus the relative difference in the RMS curvature between two samples. The fit is a sigmoidal Weibull function starting at 0.5 for zero difference and approaching 1 for large differences.

In the part of our study which addresses tactile perception, we asked participants if they perceived the micropillars on the micro-structured rubber samples in sliding touch (Fig. 4a). The probability of perceiving the pillars increases with increasing diameter of the micropillars. For equal diameter, shorter pillars are perceived with higher probability.

Participants were also asked to rank triplets of randomly rough plastic samples with respect to roughness. The psychometric curve in Fig. 4b reports the probability that participants have ranked the sample with higher rms curvature as rougher in pairwise comparison between samples. From the level of 75% probability in the psychometric curve, we extract a just noticeable difference of 0.15, i.e. differences of 15% in rms curvature (Weber fraction) are regularly detected as differences in roughness by participants. The average success rate in comparing roughness with respect to the rms curvature was 90 ± 8%. As there is rather little variation between participants in this high success rate, no correlation of individual success rates with physiological predictor parameters can be reported.

### Principal component analysis

We performed principal-component analysis (PCA) first for the outcome variables of friction and perception and then for the parameters of skin physiology. Results for the PCA of friction and perception are presented in Table 1. A KMO score of 0.912 indicated a marvelous sampling adequacy and Bartlett’s test of sphericity (*χ²* = 1009.4, *p* < 0.001) showed that the observed correlations between ratings are meaningful.

**Table 1:** Principal components in a rotated matrix according to the results obtained from friction, perception, and acuity. Loadings higher than 0.75 are printed in bold letters to indicate the outcome variables which are further analyzed as average quantities *µ*_*rough*_, *µ*_*fibril*_, and *S*_*perc*_.

Variable	PC1	PC2	PC3
	µ_rough_	µ_fibril_	*S*_*perc*_
µS09	**0.945**	0.240	0.056
µS02	**0.942**	0.274	0.091
µS08	**0.940**	0.268	0.122
µS07	**0.936**	0.255	0.157
µS03	**0.936**	0.258	0.144
µS01	**0.924**	0.234	0.184
µS150/150	0.665	0.602	0.050
µS100/100	0.658	0.476	0.151
µS150/350	0.150	**0.887**	− 0.126
µS100/200	0.308	**0.871**	− 0.048
µS100/300	0.248	**0.869**	0.037
µS40/120	0.326	**0.811**	− 0.032
Two points	0.057	− 0.048	**0.898**
Av. Percep. Pillar	− 0.282	0.062	**− 0.798**

We found three principal components, which together explain a variance of 86.1% in the data. The first rotated component (46.5% of variance) has similar high loadings between 0.92 and 0.95 for friction results of all six randomly rough plastic samples. Only friction results for the two micro-structured rubber samples with the lowest aspect ratio of 1 presented a loading of 0.66 in this component, while all other results had a loading below 0.33. We conclude that the friction coefficient measured on the six randomly rough samples is one component in our results.

The second rotated component (28.2% of variance) is dominated by friction results from the four micro-structured rubber samples with aspect ratio higher than 1, with loadings between 0.81 and 0.89. Micro-structured rubber samples with aspect ratio 1 contribute less (loading of 0.60 and 0.48) and other predictor variables contributed below 0.3. This component can be best described as the friction coefficient measured on the four samples which carry compliant fibrillar micro-structures. The third rotated component (11.4% of variance) represents the perception of micropillars and the acuity in the two-point discrimination test.

Based on this component analysis, we decided to limit the further analysis of individual outcome variables to three combined results which are defined by loadings larger than 0.75 in each principal component: the average individual friction $\:{\mu\:}_{rough}$ of six randomly rough samples, the average individual friction $\:{\mu\:}_{fibril}$ for the four compliant micro-structured rubber samples with aspect ratio higher than 1, and the individual perception sensitivity $\:{S}_{perc}$ calculated as average of the z-score for the perception of micropillars and the negative z-score for the two-point discrimination distance.

**Table 2:** Principal components in the results for skin physiological properties and age. Loadings higher than 0.65 are printed in bold letters to indicate the outcome variables which are further processed.

Variable	PC1	PC2	PC3
	Age/*MC*s	Elasticity	Deformability
*MC*s per mm^2^	**0.896**	− 0.115	0.060
Age	**− 0.880**	− 0.095	− 0.051
R2	0.150	**0.772**	− 0.150
SC thickness	− 0.084	**0.685**	− 0.238
Ridge distance	− 0.359	0.497	0.092
SC hydration	0.218	0.547	**0.693**
SG per mm^2^	0.234	0.234	**− 0.692**
R0	0.186	-0.193	**0.680**

The second PCA on physiological skin parameters and age is presented in Table 2 and reveals three principal components with a cumulative sum of explained variance of 63.9%. The first rotated component (23.4% of variance) combines age and the number of MCs per mm^2^. The second rotated component (21.6% of variance) is dominated by results for the elasticity of skin (*R2*) and the *SC thickness.* The third rotated component in this analysis with a variance of 18.9% comprises the deformability of the skin (*R0*) and the *SC hydration*. Bartlett’s test of sphericity (χ^2^ = 84.5, *p* < 0.001) shows that the observed correlations are meaningful. However, the KMO score of 0.512 indicates a barely acceptable sampling adequacy. This result suggests that partial correlations between the physiological variables and age do not allow to assign them to a smaller number of underlying meaningful components. We hence consider the physiological parameters and age in the following analyses as separate variables to predict individual differences in friction and perception.

### Correlation analysis

Based on the determination of relevant components, we calculated pairwise correlation coefficients for three outcome results and six predictor parameters (see matrix S2 in the SI). The outcome results are the average coefficient of friction *µ*_*rough*_ for the randomly rough plastic samples, the average coefficient of friction *µ*_*fibril*_ for the micro-structured rubber samples with aspect ratio higher than one, and the z-score *S*_*perc*_ for the perception sensitivity. The predictor parameters are *SC hydration*, skin deformability (*R0*) and its fast elastic response component (*Ue*), skin elasticity (*R2*), *SC thickness*, and the density of Meissner corpuscles (*MCs density*). None of these predictor parameters and none of the outcome results shows significant difference between female and male participants of our study. Throughout this section we report correlations only if the significance test against 0 resulted in a *p*-value *p* < 0.05, with few exceptions where we explicitly report non-significant correlations as tendency.

The average coefficient of friction for the randomly rough plastic samples is positively correlated with *SC hydration* (*R* = 0.75, *p* < 0.001), but also with the *SC thickness* (*R* = 0.46, *p* < 0.001) and the skin mechanical properties (*U*_*e*_: *R* = 0.46, *p* < 0.001; *R0*: *R* = 0.38, *p* < 0.004; *R2: R* = 0.47, *p* < 0.001). Note that the correlation is numerically stronger for the fast elastic response *U*_*e*_ than for the overall deformability *R0.* Since the fast elastic deformation *U*_*e*_ in the first 100 ms is part of the total deformation R0, we confirmed that the delayed deformation (R0-Ue) occurring between 100 ms and 2 s is not correlated with friction. We conclude that only the fast component *U*_*e*_ of deformability is relevant for friction variation and, therefore, use only *U*_*e*_ but not *R0* to represent deformability in the further analyses.

The average coefficient of friction for the micro-structured rubber samples with aspect ratio higher than one only weakly correlated to the *SC hydration* (*R* = 0.20, *p* = 0.1) but is more strongly correlated to the deformability of the skin (*U*_*e*_: *R* = 0.43, *p* < 0.001) and to the *SC thickness* (*R* = 0.33, *p* = 0.01).

**Fig. 5:**
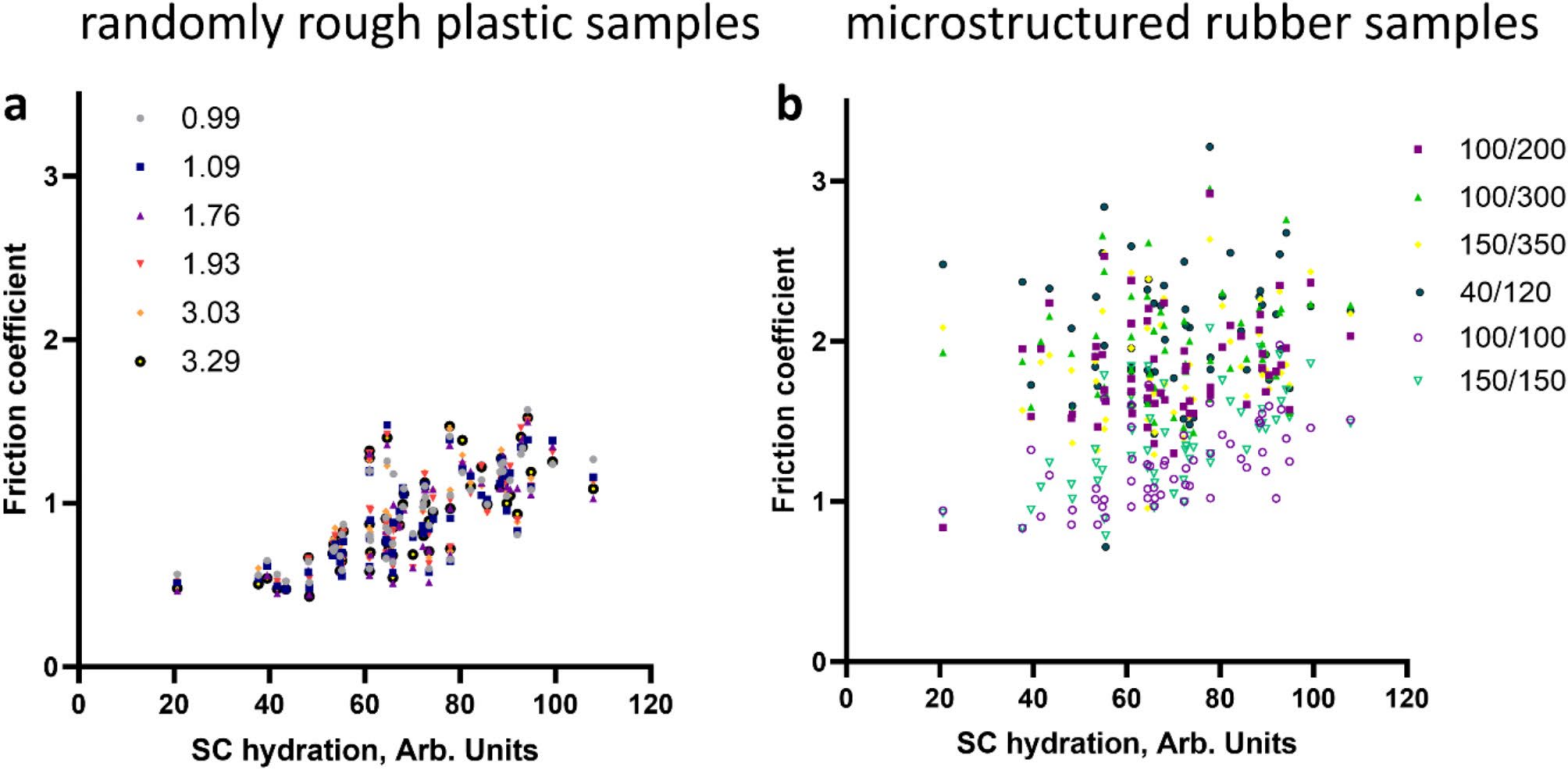
Friction coefficients as function of the *SC hydration*. (**a**) Results for the randomly rough plastic samples. The legend indicates the rms curvature of the surfaces in mm^−1^. (**b**) Results for the micro-structured rubber samples. The legend indicates diameter and height of the micropillars. Full symbols indicate samples with aspect ratio larger than one, hollow symbols samples with aspect ratio of one. Note that the figure contains friction values for each sample and individual, whereas in the multiple regression only individual averages could be predicted.

For further insight into the role of skin physiology for friction, we performed multiple linear regression analyses on the outcome results, using the predictor parameters *SC hydration*, *U*_*e*_, R2, *SC thickness*, and *MC* density. For the randomly rough plastic samples, the regression explained 74% of the variance in the average friction coefficients. *SC hydration*, *SC thickness*, deformability *U*_*e*_, and elasticity R2 contribute significantly to the multilinear model. The parameters are ordered here by decreasing relevance as indicated by the decreasing *t* values of the regression (see Table 3 for numerical values). The important role of *SC hydration* for predicting the friction coefficient can be recognized by the trend in Fig. 5a. For the micro-structured rubber samples with aspect ratio higher than one, regression explains a variance of 34% of the variance in the average friction coefficients. Only the deformability *U*_*e*_ and the *SC thickness* contribute significantly to the linear model. The lack of a dependence of friction on *SC hydration* can be recognized in Fig. 5b.

**Table 3:** Significance of multiple linear regression (degrees of freedom 48).

	*R* ^2^	SC hydration		U_e_		R2		SC thickness		MC density	
		t	*p*	t	*p*	t	*p*	t	*p*	t	*p*
µ_rough_	0.74	5.3	3.3E−6	3.2	0.003	2.4	0.02	3.8	3.5E−4	1.2	0.2
µ_fibril_	0.34	− 1.3	0.18	3.9	3.2E−4	0.9	0.4	2.9	0.006	1.0	0.3
S_perc_	0.40	2.0	0.05	− 0.5	0.6	− 1.0	0.3	-0.4	0.7	4.9	1.0E−5

**Fig. 6:**
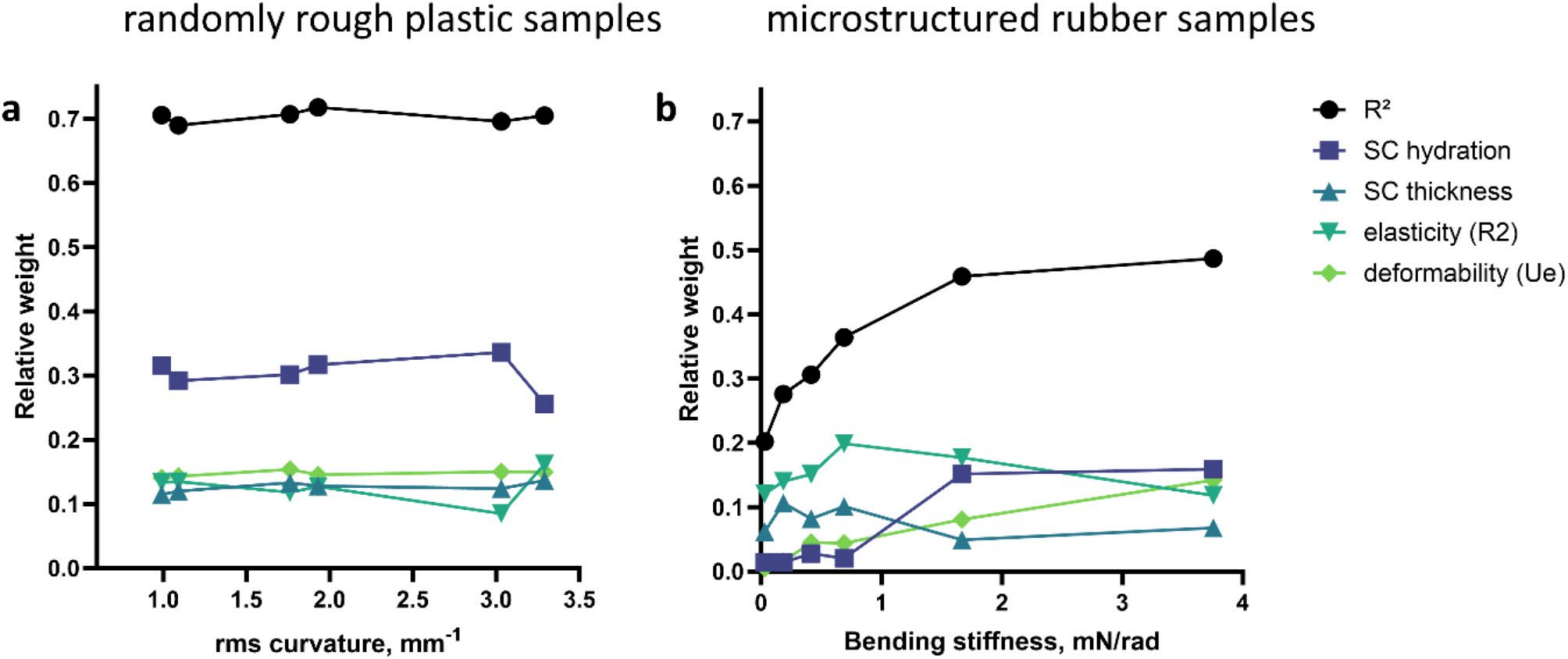
Variance in the coefficient of friction µ which can be explained by the physiological parameters *SC hydration*, fast elastic response *U*_*e*_, elastic recovery R2, and *SC thickness* and their relative weights. (**a**) Randomly rough plastic samples described by their rms curvature. (**b**) Micro-structured rubber samples characterized by the bending stiffness of pillars. Black circles indicate the total explained variance in friction, color symbols provide the relative weight of each parameter in the explained variance.

We were also interested in the variation of friction between participants for each single sample. Repeating the multiple linear regression on *SC hydration*, *U*_*e*_, *R2*, and *SC thickness* for each of the six randomly rough plastic samples explains close to 70% of variance in the friction coefficient (see Fig. 6a), almost as much as for the average over all samples. To identify the relative importance of the partially correlated predictor parameters, we calculate their relative weight in the explanation of variance and show the results in Fig. 6a. The *SC hydration* is with close to 30% about double as important as the deformability *U*_*e*_ (close to 15%) and then the *SC thickness* or elasticity *R2* (both 12–13%).

The findings are different for each of the six micro-structured rubber samples. The variance in the friction coefficient, which can be explained by the same four physiological predictor parameters, depends on the geometry of the micropillars, more precisely on their bending stiffness (see Fig. 6b). While only 22% of the variance can be explained by physiological parameters for the pillars with the lowest bending stiffness (40/120), 49% can be explained for the pillars with the highest bending stiffness (150/150). The relative weights of the predictor parameters reveal that the importance of the skin deformability *U*_*e*_ increases from zero for the most bendable pillars to 14.2% for the least bendable ones. Similarly, the *SC hydration* plays almost no role for bendable pillars but explains 15% of the variance in friction for stiffer pillars with aspect ratio 1. This finding can be recognized in Fig. 5, where the data point for samples with aspect ratio 1 (open symbols) show less scatter and a clear dependence on *SC hydration*. The importance of skin elasticity and *SC thickness* have less obvious tendencies, but the skin elasticity *R2* is more important than the *SC thickness* for all samples.

The individual friction coefficients of participants are strongly correlated ($\:R\ge\:$ 0.94) between each pair of randomly rough plastic samples (see matrix S3 in SI). Friction on one sample as predictor explains thus more variance ($\:{R}^{2}\ge\:0.88$) in the friction of another sample than the multiple linear regression into physiological parameters summarized in Fig. 6a. We conclude that there are consistent individual differences in friction between participants, beyond the 70% variance which is explained by the four parameters *SC hydration*, fast elastic deformation *U*_*e*_, elastic recovery *R2*, and *SC thickness*. Systematic individual differences may be looked for in the exploration procedures^28^. The number of cycles per second, a rough approximation for the fingertip speed, is the one individual parameter of exploration procedures which we can add to our multiple regression analysis. There is a negative correlation of the friction coefficient with the number of cycles per second for each sample, which is however weaker than the correlation to each of the four physiological parameters. Including the cycles per second as predictor into the regression increases the explained variance slightly to values up to 72% for the six randomly rough plastic samples.

For the micro-structured rubber samples, correlation of individual friction coefficients between pairs of samples is weaker (between $\:R=0.38$ and $\:R=0.80$) than for randomly rough plastic samples (see matrix S3 in SI). One of the strongest correlations is between the two samples with the lowest bending stiffness, sample 40/120 and 100/300. Using friction of one of these two samples as predictor explains 61% of the variance in friction on the other sample, while physiological parameters explain less than 30% for either of the two. For the micro-structured rubber samples, we do not find a significant correlation between friction coefficients and cycles per second in the tactile exploration. We conclude that for micro-structured rubber surfaces with bendable fibrils, there are consistent differences in friction between participants which are only partially explained by our physiological parameters.

Finally, we report correlation for the perception sensitivity. Pairwise, the perception sensitivity *S*_*perc*_ is positively correlated with the *density of Meissner corpuscles* (*R* = 0.59, *p* < 0.001) and weakly with the *SC hydration* (*R* = 0.30, *p* = 0.02). The multiple linear regression explains 40% of the variance in *S*_*perc*_, where *MC* density and *SC hydration* contribute significantly (see Table 3). In an additional analysis, we found a significant (*p* = 0.005) negative interaction between density of Meissner corpuscles and *SC hydration*, i.e. higher hydration of the *stratum corneum* reduces the dependence of perception sensitivity on the density of Meissner corpuscles. Including the interaction term into the regression on *MC* density and *SC hydration* increases the explained variance to 47%. We would like to mention that the correlation of the perception sensitivity with age (*R* = − 0.62, *p* < 0.001, bootstrapping indicates a 95% confidence interval between $\:R=-0.39\:and\:R=-0.77$, $\:p<0.002\:$for linear fit to all 10^6^ resampled data sets) is slightly stronger than the correlation with *MC* density.

In summary, the 60 participants in this study show large relative variations in fast skin deformability and the density of *MC*s, and significant variations in *SC hydration* and *SC thickness*. The *density of MCs* decreases with age and explains the variance in tactile sensitivity, with only weak contributions from the other parameters. *SC hydration*, skin deformability, and *SC thickness* largely explain the variance in friction on randomly rough plastic samples. The situation is more complex on the micro-structured rubber samples, where physiological parameters explain only part of friction variance, particularly for the fibrillar samples with micropillars that have lower bending stiffness.

## Discussion

The results of this study contribute to understanding the influence of skin physiology parameters and material properties on finger pad friction and tactile perception. Skin microscopy, and measurements of moisture and deformation in combination with friction and subjective tactile rating data from sixty participants make this study one of the largest in terms of participant number in this field of research^9,20,29^.

The list of parameters with large relative variation among participants agrees with that of parameters with stronger correlations with finger pad friction and tactile perception: fast skin deformation, density of Meissner corpuscles, and hydration of the *stratum corneum*. On the other hand, the surprisingly small relative variation of the finger ridge distance explains the finding that this parameter does not predict the observed variations in friction or perception. We will now discuss the results for skin deformability, for friction, and for tactile perception in the light of their correlations with skin physiological parameters.

The cutometer quantifies the temporal development of skin deformation upon suction into a hole. Several parameters have been suggested to describe the extent of deformation and its relaxation and the relative contribution of fast and slow deformation^10,13^. Here we have focused on the deformability *R0* and its fast component *U*_*e*_, as well as the elasticity *R2*, which describes how much of the deformation is relaxed in the seconds after suction has ended. Other cutometer parameters which have been suggested are correlated with these fundamental parameters (see correlation matrix in SI).

To discuss its mechanical response, we can conceptualize skin as a soft material where hydration levels and the number of lamellar layers (*SC thickness*) influence its deformability and elasticity. The skin is a viscoelastic and anisotropic material^30^, with time-dependent deformation response^31^. Elasticity arises for example from bond stretching along the collagen fibers, while viscosity originates in the diffusion of molecules within porous skin layers^32^. It is surprising that the deformability of the skin does not depend on the *SC thickness*. We must keep in mind, however, that the diameter of the suction orifice is larger than the *SC thickness* and that the maximum deformation is smaller than the *SC thickness*, so that the thickness-dependent bending stiffness of the *stratum corneum* plays a minor role for the deformability as measured by the cutometer. We should rather think of the *stratum corneum* as a bendable barrier film protecting the deforming dermal layers. This picture agrees with a multilayer model of the suction experiment, which reveals that most of the deformation occurs in the dermis and probably hypodermis, with only negligible contributions from the epidermis^33^.

The measured hydration of the *stratum corneum* is positively correlated with the skin deformability under suction stress. We found that the fast deformation of the skin *U*_*e*_ in the first 0.1 s is correlated with the *SC hydration* (*R* = 0.49, *p* < 0.001), while the delayed deformation (*R0-U*_*e*_) in the time between 0.1 and 2.0 s is not. It has been suggested that increased hydration causes a softening of the *stratum corneum* by the presence of hygroscopic proteins within the corneocytes^10,34^. We conclude that the positive correlation of *SC hydration* with deformability is due to plasticization of the *stratum corneum* which affects the immediate deformation, while the delayed deformation originates in liquid flow within the lower-lying dermal tissue and cannot be predicted by measurements of the *SC hydration*. The effect of hydration on skin deformability has been enhanced with moisturizing creams and was attributed to a change in *stratum corneum* modulus^35^.

We will start the discussion of friction with results for the randomly rough plastic samples. *SC hydration* was the single parameter which explained most of the variation in friction coefficients. The trend is clearly observed in Fig. 5a. It is interesting to note that *SC hydration* has a higher correlation to friction than skin deformability or *SC thickness*. We conclude that *SC hydration* has a direct effect on friction, rather than contributing to friction by modulation of mechanical skin properties. The most probable mechanism is capillary condensation of water or sweat between the skin and roughness asperities^36,37^. Skin deformability, elasticity, and *SC thickness* carry only half of the relative importance in explaining variance in friction compared to *SC hydration*.

It is surprising that friction on the rough samples depends rather weakly on the skin deformability. The weak correlation can be explained by considering that deformation measurements with the cutometer address length and time scales which do not fully match the scales relevant for friction on the rough plastic samples. The curvature produced by the cutometer during the skin suction is about 0.33 mm^−1^ and thus up to ten times smaller than the rms curvature of the surfaces. The time scale of deformation in the cutometer is 0.1 s for the fast elastic response and about 2 s for the delayed component, while the characteristic time scale for friction on rough samples can be estimated as the finger ridge distance divided by the sliding velocity to be less than 0.01 s. The necessity to shift the mechanical characterization of the skin towards faster time scales is highlighted by our finding that the friction coefficient for randomly rough plastic samples is correlated with the fast elastic response, but not with the delayed component. Unfortunately, dynamical mechanical characterization of the skin with higher bandwidth and at smaller length scale is a non-trivial experimental task^38^.

The dependence of friction on *SC thickness* is also rather weak. The *SC thickness* affects the elastic recovery of skin but not the overall deformability. Corniani and collaborators have shown that the indented *stratum corneum* stretches, compresses, and undergoes considerable shearing orthogonal to the skin surface, but limited horizontal shearing^19^. Taking both observations together, we suggest that the *stratum corneum* is a rather passive mechanical element which transmits shear stress to the dermal layers but does not dissipate friction shear itself.

Skin physiological parameters explain the variance in friction coefficients among participants for each randomly rough sample similarly to a high fraction of 70%, compared to the 46% explained by a multivariate model for the dynamic friction coefficient including the physiological parameters skin temperature, age, and height^29^. The strong correlation ($\:{R}^{2}\ge\:0.86$) of friction coefficients between samples shows that there are systematic differences in friction between participants which are not explained by the measured physiological parameters. These differences could be caused by systematic variation of the real contact area between skin and surface at a given load, due to individual differences in the shape of the finger pad and the structure of the ridges^39^. The differences in friction could also be caused by individual tactile exploration procedures. The only parameter describing the exploration procedure which we can assess is the number of circles per second performed by the participants, which adds little to the explained variance. Friction coefficients are also sensitive to the angle between distal phalange and sample surface, probably an effect of the real contact area^40^. While we asked participants to use their straight index finger, the actual implementation of this task may have varied systematically between participants.

The friction coefficients show a weak positive correlation with rms curvature of the surfaces of plastic samples, which is a measure for the roughness at the scale of finger ridges. Adhesion and deformation contribute to dissipative friction. Adhesive friction decreases with decreasing real contact area when moving from flat surfaces to rougher ones^41,42^. Deformation friction increases when higher roughness asperities cause more viscoelastic deformation of the skin^37^. Finger pad friction on our set of randomly rough samples follows the deformation mechanism, in agreement with a previous study^43^. The deformation mechanism is also in agreement with the correlations found between friction coefficients and the mechanical parameters *SC thickness*, deformability, and elasticity. While the influence of roughness on friction is weak, participants successfully ranked the roughness after tactile exploration.

We will now turn our discussion to friction on the micro-structured fibrillar samples. The aspect ratio of the micropillars, i.e. the relation of their height to their diameter is a significant predictor for friction coefficients. This systematic dependence can be recognized in the plot in Fig. 5b. Friction increases with higher aspect ratio, and this effect has previously been attributed to the lower bending stiffness of taller micropillars, which are bent into larger contact with the finger pad skin^7,44^.

Friction on micropillar arrays with aspect ratio 1 (100/100, 150/150) has not only the lowest coefficients. It is also correlated with *SC hydration* (see hollow symbols in Fig. 5b) like on the randomly rough plastic surfaces. The similarity of friction results for rubber samples with sturdy micropillars of aspect ratio 1 and for randomly rough plastic samples is manifest in the absolute values of the friction coefficient, the dependence on *SC hydration*, and the communality in the first component of the PCA (see Table 1). We conclude that the structuring of rubber samples with arrays of short pillars with high bending stiffness has a similar effect on friction as roughness on the plastic samples. Structuring the surface with more compliant micropillars of higher aspect ratio leads to an increase in friction which does not depend anymore on the *SC hydration*.

For the micro-structured rubber surfaces, skin physiological parameters explain much less variance in friction between participants than for the randomly rough plastic samples. Furthermore, the lower the bending stiffness of the micropillars, the smaller the relevance of skin physiology for the strength of friction. The relative importance of skin deformability for predicting friction is increasing with increasing bending stiffness of the micro-pillars, indicating that only the stiffer pillars deform the skin and contribute to friction by deformation. Similarly, *SC hydration* carries significant relative weight in the explanation of friction variance only for the stiffer pillars. We suggest that capillary condensation occurs between skin and the flat top surfaces of unbent pillars but not between skin and the curved side walls of bent pillars (see picture in Fig. 3a). The larger role of *SC hydration* for friction on the randomly rough surfaces compared to the micro-structured rubber can in part be explained by stronger capillary condensation on the more hydrophilic plastic.

Between 50 and 80% of the variance in friction on the different micro-structured rubber samples cannot be explained by the physiological parameters. Consistent differences in the tactile exploration procedures between participants may lead to different forms of bending the pillars into contact with the finger ridges and to differences in the contact area. Tactile exploration procedures may thus explain a larger fraction of the variance as indicated by the stronger correlation of friction coefficients between samples with equal aspect ratio ($\:{R}^{2}\ge\:0.58$). Finally, the flexible micropillars may offer more degrees of freedom to dissipate energy in the sliding contact by adhesion and deformation, leading to more unexplained random scatter in the measured friction coefficients than in the case of the randomly rough plastic samples.

Our measure for perception sensitivity $\:{S}_{perc}$ is the component composed of two-point discrimination acuity and of the sensitivity to detect the pillars on micro-structured rubber samples. This tactile sensitivity has a positive correlation with the density of Meissner Corpuscles. A higher density of mechanoreceptors has previously been suggested as an explanation for the finer tactile acuity of smaller fingers^27^. Meissner corpuscles (*MC*s) are rapidly adapting receptors located closer to the skin’s surface than other mechanoreceptors^45^. *MC*s are less sensitive to static than to dynamic forces and primarily detect skin slip^46^. It is believed that the dynamic skin deformation during active exploration deforms the *MC*s, triggering the perception of fine structures^47^. The density of pillars on the micro-structured rubber surfaces ranges from 180 mm^−2^ to 12 mm^−2^. Pillars with a density much higher than that of *MC*s (7.2 mm^−2^ on average) are not sensed by participants, but those with comparable densities are detected in sliding touch. We suggest that the interaction of bent pillars with finger ridges may elicit the neural correlate of microslips, leading to an effective perception of these fibrillar surfaces if the density of pillars is of the same order as that of Meissner corpuscles. Since it is generally assumed that *MC*s provide less spatial resolution compared to slowly adapting mechanoreceptors^46,48^, a contribution of other mechanoreceptors to the perception of pillars via skin indentation or vibration must be considered.

Although we found no significant correlation between *SC thickness* or *SC hydration* and perceptual sensitivity, a role of skin morphology for tactile perception has been discussed. Dynamic contact forces result in the mechanical deformation of the skin structure. Corniani et al. suggest that the neural responses do not follow the bending of ridges itself, but rather the deformation and movement of the ridge flanks^19^. The distribution of ridges is then adapted to detect the strain gradients, and the arrangement of finger pad ridges provides potentially a better spatial resolution than an individual ridge alone. The position of *MCs* close to the ridges, as observed in Fig. 1d, supports the effective translation of shear stresses into neural stimuli in active exploration. It should be noted that the absence of any correlation between ridge distance and perceptional sensitivity in our data does not imply that ridges play no role in tactile perception. The ridge distance is the parameter with the smallest relative variation among participants and can, therefore, hardly offer any correlations.

Ageing reduces the density of *MCs*, in agreement with previous reports^15,16^. The correlation of perception sensitivity with age is slightly stronger than its correlation with the density of *MCs*. It was predicted that ageing-related structural changes to the skin reduce the proportion of stimuli meeting the receptor amplitude detection threshold^49^. Furthermore, participants above an age of 50 years tend to exhibit a hyperkeratinization process in the remaining corpuscles^50^, which could also affect their capacity to discriminate small structures. It was also suggested that the decrease in tactile acuity was related to an increase in finger pad size for elderly^9^. Finally, we note that more mechanoreceptors than *MC*s are involved in tactile sensitivity and that a decrease for example of Merkel cells with age may contribute to the decay of tactile sensitivity^26^. The observed interaction between *MC* density and *SC hydration*, which increases the explained variance in perception sensitivity significantly to 47%, supports suggestions that hydration is a key element in preserving tactile sensitivity for the elderly^20,51^.

Participants succeeded in discriminating roughness of the plastic samples when the relative difference in curvature was more than 15%. Sahli et al. studied the perceived similarity between samples of the same set upon sliding touch and found that differences in surface curvature of more than 0.8 mm⁻¹ were relevant for the participants’ judgements^43^. The discrimination of roughness by participants in this study was better by a factor of two, as the differences in surface curvature ranged from 0.67 to 2.3 mm⁻¹. The difference may be due to the different task, which was addressing roughness comparison directly in this study but perceived similarity in the earlier study. The smallest wavelength on the randomly rough plastic surfaces is 500 μm, more than the average distance between Meissner corpuscles (Fig. 1d). We did not find a significant correlation between the density of *MC*s and the average success rate of participants in ordering samples with respect to roughness, partially because the success rate was higher than 90% for most participants. Libouton and collaborators already observed that finger pad sensitivity is not necessary for tactile roughness discrimination^52^. These findings support the notion that roughness discrimination is result of an interplay of spatial and temporal responses of all classes of mechanoreceptors which generate a broad spectrum of neural correlates within the somatosensory cortex^53^. The capability of in vivo microscopy to quantify only the density of *MCs* while disregarding other mechanoreceptors or nerves limits a comprehensive understanding and prediction of roughness discrimination. This gap may be partially filled by additional physical measurements of roughness-induced vibrations and individual finger pad transfer functions^54^.

## Summary

We studied friction and tactile perception for 60 participants on samples from two distinct classes of materials, namely randomly rough hard plastic samples and micro-structured fibrillar elastic rubber samples. Systematic variation of parameters for these characteristic surface structures allowed for correlation with those physiological parameters of the finger pad skin which can be measured non-invasively by laser microscopy, hydration detection, and deformation.

The variation of finger-pad friction on rough plastic surfaces between participants can be explained largely by skin physiology, most prominently by the moisture in the *stratum corneum* which contributes to frictional interaction by the formation of capillaries between skin and roughness asperities. For both materials, the fast (0.1 s) component of skin deformability is more relevant than the overall deformability, indicating a need for improved experimental characterization of skin deformation at faster time scales. Friction variations are less well explained by physiology for the fibrillar samples when the micropillars are soft and bendable. There are systematic differences between participants in friction which are not explained by the measured physiological parameters, more for the bendable fibrils than for the rough plastic. We suggest that details of the individual tactile exploration procedures cause variations in the interaction of finger ridges and bendable fibrils.

Spatial tactile perception sensitivity decreases with age in negative correlation and increases with the density of Meissner corpuscles. Well hydrated skin reduces the dependence of sensitivity on the density of Meissner corpuscles. The results suggest that *stratum corneum* hydration, skin deformability, and age are important factors for friction and perception in active tactile exploration of materials, and that the roles of skin morphology and individual exploration strategies deserve further attention.

## Methods

### Participants

Sixty healthy volunteers (23 men, 37 women) with an age ranging from 20 to 70 years (average 34, standard deviation 11; divided in groups of 15 in the following distribution: 20–27, 28–30, 31–33, and 34–70) were enrolled in this study. This study was approved by the Ethics Committee of the Charité –Universitätsmedizin Berlin (EA1/114/22) and performed in accordance with the ethical standards of the Declaration of Helsinki, as revised in 2013. All participants gave their written informed consent to participate in this research.

### Apparatus

*Stratum corneum* hydration (*SC hydration*) was measured by a capacitance method (Corneometer^®^ TM CM 825, Courage & Khazaka Electronic GmbH, Cologne, Germany). The morphology of the skin was evaluated by Laser Scan Microscopy (LSM, VivaScope 1500 Multilaser, MAVIG GmbH, Munich, Germany). VivaBlock^®^ images were acquired in an area of 3 mm × 3 mm and analyzed to determine the density of sweat glands and ridge distances in a depth of 50 to 75 μm and the density of Meissner corpuscles at the dermal-epidermal junction, whose depth varied between participants. Optical coherence tomography (OCT, VivoSight Michelson Diagnostics Ltd., Maidstone, UK) was applied to determine the thickness of the *stratum corneum* (*SC thickness*).

Mechanical properties of the skin were measured with a cutometer (MPA580, Courage & Khazaka Electronic GmbH, Cologne, Germany). The handheld probe of this device is brought into contact with the finger pad skin. By applying a vacuum of 450 mbar, the skin is sucked into an orifice with 2 mm diameter for 2 s and then released again for 2 s. The displacement of the skin in the center of the orifice is measured optically. The cycle of deformation and relaxation is repeated ten times. Three deformation parameters are analyzed in this study, the immediate displacement within 0.1 s of starting the vacuum suction (*U*_*e*_) as a measure for the fast elastic response of the skin, the total displacement within 2 s of vacuum (R0) as measure for the deformability, and the relative relaxation (R2) 5 s after the vacuum is released as measure for the elasticity. These parameters have been defined and attributed in previous publications^10,13^.

For the two-point discrimination experiment, participants were instructed to insert their hands into a black box, thereby eliminating visual contact with the apparatus. Two sharp plastic tips with adjustable distance (Exacta, North Coast Medical Inc.) were brought into gentle contact with the finger pad. Participants indicated whether they perceived one or two distinct contact points. A random order of distances 0, 2, 3, 4, 5, 6, 7 and 8 mm was tested for each participant, and the minimal distance perceived as separated points was tested again together with the 1 mm distance. The minimal distance perceived as separated points was recorded. Lower values indicate higher spatial acuity^17^.

For the tactile exploration experiments, samples were presented in a black box with an opening for the exploring hand to avoid visual information in the perceptional tasks. Participants were informed about the position of each sample by indicators in front of the box. Participants wore sound-proof headphones to avoid auditory perception related to tactile exploration. Participants explored the sample sets in randomized order. They were instructed to perform ten circles with the straight index finger to ensure that the center of the finger pad is in contact with the sample.

For the friction measurements, the samples were mounted on a 3-axis force sensor (K3D120 with GSV-8 amplifier, ME-Messysteme, Germany). Forces in the normal direction ($\:{F}_{N}$) and friction forces ($\:{F}_{F}=\sqrt{{F}_{x}^{2}\:+\:{F}_{y}^{2}}$ ) were recorded at a sampling rate of 50 Hz. The friction coefficient ($\:\mu\:$) was calculated as the ratio $\:{F}_{F}/{F}_{N}$. We report the median friction coefficient during touch in each trial, where touch is assumed when the normal force exceeds four times the noise of the unloaded force sensor. The investigator provided guidance to the participants to adjust the applied normal force within the range of 0.8 to 1.2 N. Due to temporal problems with data recording, we had to omit the data of two participants and of seven trials from other participants, i.e. we analyzed 689 out of 720 measurements (96%). The participants touched each sample between 15.4 and 69.4 s and performed between 1 and 26 circles. The mean circle rate was 0.4 circles per second (minimum 0.06 1/s, maximum 1.21 1/s).

### Stimuli

The two sample sets for tactile exploration and perceptional tasks were made from two different materials. Each of the twelve samples measured 50 mm by 50 mm. The first sample set was made of an elastic, rubbery material (polyurethane, elastic modulus $\:E\:=\:1.7$ MPa, contact angle with a sessile drop (4 µl) of water on flat side 83°, details of sample production in Fehlberg et al.^7^) The surface microstructure was a hexagonal array of micropillars with flat tops and a center-to-center distance of twice the diameter. Diameter $\:D$ and height $\:L$ for the six samples were 40/120, 100/100, 100/200, 100/300, 150/150 and 150/350, values given in µm. It was reported previously that the tactile perception of similarity between these samples is dominated by the bending stiffness of the micropillars^44^ and that participants can correctly judge differences in finger pad friction between samples^7^. The bending stiffness, i.e. the lateral force $\:{F}_{L}$ which must be applied to the top of a pillar to bend it into an angle $\:\theta\:$ is $\:{F}_{L}/\theta\:=\pi\:E{D}^{4}/32{L}^{2}$. We will refer to these samples as micro-structured rubber samples.

The second set of samples was made from a hard plastic (polyacrylate, contact angle with water on flat side 71°). The samples were produced by 3D printing with mathematically defined randomly rough surfaces (for details see Ref^43^). Each sample had the same overall root-mean square (RMS) roughness of *Sq* = 0.4 mm at the scale of the finger pad. However, the decay of roughness amplitude towards smaller length scales, described by the Hurst roughness exponents of *H* = 0.4, 0.6, and 0.8, varied between samples. Therefore, the roughness varied between samples at the scale of the finger ridges. We quantify the difference of roughness at small length scales by the RMS curvature of the six surfaces, i.e. by the square root of the average square of the second derivative of the height function (3.29, 3.03, 1.93, 1.76, 1.09, 1.00 mm^−1^). It was found that roughness at small length scales is the key parameter for finger pad friction and the tactile perception of similarity between these samples^43^. We will refer to these samples as randomly rough plastic samples.

### Design and procedure

Physiological skin parameters were measured in non-invasive in-vivo experiments to reveal their relationship with friction and tactile perception. Hydration of the *stratum corneum* (*SC hydration*), skin deformability (*U*_*e*_, *R0*) and elastic recovery (*R2*), thickness of the *stratum corneum* (*SC thickness*), and density of Meissner corpuscles (*MC density*) as those mechanoreceptors, which are accessible by non-invasive optical imaging, were determined in the center of the finger pad of the index finger’s distal phalange. To assess the spatial acuity of tactile perception, we conducted a two-point discrimination test in which participants indicate the minimum distance on the skin at which they perceive two point-like stimuli as separate.

For the first sample set of micro-structured rubber samples, we presented each sample separately in random order and asked participants to explore it with their circular motions of their straight index finger. During this tactile exploration, we measured the applied normal forces and the friction forces. As perceptional task we asked: “During your tactile exploration, could you feel the presence of pillars in this sample?” Prior to the explorations, an enlarged photograph of the micropillars was presented to the participants to clarify the explored structure.

For the second set of randomly rough plastic samples, the six samples were grouped into ten unique triplets and presented to each participant. The sample order was randomized between participants. The task was phrased as follows: “Please explore the surfaces with circular motions of your straight index finger and, after that, determine the order of roughness of the samples, from the least rough to the roughest.” The participants evaluated the samples only once and always followed a left-to-right sequence. During tactile exploration, we measured the applied normal forces and the friction forces. Each sample appeared five times in the ten triplets, resulting in ten pairs of samples which were compared within a triplet.

The participants were instructed to not apply any cosmetic formulation such as moisturizing lotions to their hands for at least 24 h prior to the study. All experiments were carried out at standard conditions (humidity 40–50% and temperature 22–25℃) from September to November 2023 in Berlin, Germany. The study was performed in the following order: acclimatization and explanation of the study; two-point discrimination test; tactile exploration of micro-structured rubber samples and decision whether micropillars were perceived; measurement of *SC hydration* and skin mechanical properties; tactile exploration of randomly rough plastic surfaces and ranking of their roughness; optical coherence tomography and laser scanning microscopy of the skin structure. The duration of the study was 1.5–2 h per participant.

### Data analysis

Mean values are presented ± the standard deviation. Relative standard deviations were calculated as the standard deviation divided by the mean of that distribution. The *z* scores of distributions were calculated by subtracting the mean from all values and dividing them by the standard deviation. Correlations are quantified by the Pearson *R* coefficient, their significance is tested by ANOVA and indicated by the *p* value for the Null hypothesis of no correlation. For bootstrapping of correlations, the data sets were resampled 10^6^ times by drawing values pairs with replacement. Confidence intervals for the results contain 95% of the respecting fit metric across resampled data sets. Results of multiple linear regression are quantified as explained variance $\:{R}^{2}$, the significance of each parameter is indicated by the *t* statistics with its degrees of freedom, and the probability *p*. Distributions and correlations were analyzed using the software Origin 2022 (OriginLab Corporation), bootstrapping was implemented with the *bootstrap (n_resamples = 100000*,* confidence_level = 0.95)* function in SciPy (1.13.1). The Weibull function in Fig. 4b is given as $\:y=1-0.5\cdot\:\text{exp}\left(-{\left(kx\right)}^{d}\right).\:$The parameters do not carry any physical meaning, as the curve is fitted only to predict the just-noticeable difference in roughness at a probability of 0.75.

To determine the relative importance of correlated predictor parameters in multiple linear regression, we calculate their relative weights based on Johnson’s method^55^ using a web-based tool^56^. To identify communalities between experimental results across participants, we performed a principal component analysis (PCA) on the individual data using the Kaiser-criterion and varimax-rotation of the components. We also checked whether the data are suitable for a PCA using the Keyser-Meyer-Olkin (KMO) value and Bartlett’s test of sphericity. We calculated the individual component scores of each material in the stimulus set with Bartlett’s method. PCA was performed using IBM SPSS Statistics 28.

## Electronic supplementary material

Below is the link to the electronic supplementary material.

Supplementary Material 1

## Data Availability

A table with all numerical results used for PCA and correlation analyses is published as Infante, V.H.P; Fehlberg M.; Saikumar, S.; Drewing K., Meinke, M.C., Bennewitz, R. (2024). Data set for manuscript “Skin factors in tactile friction and perception of materials: hydration, deformability, and age” [Data set]. Zenodo. https://doi.org/10.5281/zenodo.12806178. Underlying raw data and images are available from the corresponding author upon reasonable request.
