# Supplementary material for: The role of skin hydration, skin deformability, and age in tactile friction and perception of materials
Source: Sci Rep. 2025 Mar 22;15:9935. doi: 10.1038/s41598-025-95052-4 (PMC11929835; doi:10.1038/s41598-025-95052-4)
Supplement: Supplementary file 1 — Supplementary Material 1 [file 41598_2025_95052_MOESM1_ESM.pdf]

Supplementary Information on
“Hydration, deformability, and age are skin factors in tactile friction and perception of materials”

Victor H. P. Infante[1,2], Maja Fehlberg[1,3], Sairam Saikumar[1,3], Knut Drewing[4], Martina C. Meinke[2], Roland Bennewitz[1,3,*]

[1]INM - Leibniz Institute for New Materials, Saarbrücken, Germany

[2]Charité – Universitätsmedizin Berlin, Corporate Member of Freie Universität Berlin and Humboldt Universität zu Berlin, Department of Dermatology, Venereology and Allergology, Center of Experimental and Applied Cutaneous Physiology, Berlin, Germany

[3]Saarland University, Department of Physics, Saarbrücken, Germany

[4]Department of Psychology, Justus Liebig University, Giessen, Germany

Table S1 – Skin parameters differentiated by sex. A *p* value is provided for the probability of the Null hypothesis that there is no difference in mean value of the distributions (*t* test).

	Female	Male	*p* value
**SC hydration**	72 ± 15	70 ± 23	0.67
**SC Thickness**	255 ± 63	275 ± 70	0.21
***MCs* per mm^2**	7.6 ± 3.3	6.7 ± 2.2	0.32
**SGs per mm^2**	5.7 ± 1.2	5.0 ± 1.2	**0.034**
**Two-point discrimination**	2.8 ± 0.7	2.8 ± 0.9	0.90
**Age**	34 ± 12	33 ± 9	0.62
**R0**	0.15 ± 0.04	0.14 ± 0.05	0.47
**R2**	0.74 ± 0.06	0.72 ± 0.08	0.46
**Ridge distance**	429 ± 28	488 ± 66	**0.0006**

Table S2 – Correlation matrix for predictor parameters and average outcome values.

	SH	SC Thickness	Ue	R0	R2	MCs per mm^2	SGs per mm^2	RD	Age	μ_fibril	μ_rough	Sperc
SH	1.00	0.12	0.49	0.40	0.31	0.14	0.06		-0.31	0.20	0.75	0.30
SC Thickness	0.12	1.00	-0.09	-0.11	0.39	-0.13	-0.17	0.18	0.04	0.33	0.46	-0.13
Ue	0.49	-0.09	1.00	0.97	-0.14	0.22	0.04	-0.15	-0.20	0.43	0.46	0.21
R0	0.40	-0.11	0.97	1.00	-0.21	0.17		-0.19	-0.17	0.39	0.38	0.19
R2	0.31	0.39	-0.14	-0.21	1.00		0.05	0.09	-0.13	0.15	0.47	-0.04
MCs per mm^2	0.14	-0.13	0.22	0.17		1.00	0.40	-0.27	-0.69	0.16	0.19	0.59
SGs per mm^2	0.06	-0.17	0.04		0.05	0.40	1.00	-0.66	-0.17	-0.07	-0.04	0.05
RD		0.18	-0.15	-0.19	0.09	-0.27	-0.66	1.00	0.17	-0.07	0.01	-0.13
Age	-0.31	0.04	-0.20	-0.17	-0.13	-0.69	-0.17	0.17	1.00	-0.13	-0.36	-0.62
μ_fibril	0.20	0.33	0.43	0.39	0.15	0.16	-0.07	-0.07	-0.13	1.00	0.50	-0.04
μ_rough	0.75	0.46	0.46	0.38	0.47	0.19	-0.04	0.01	-0.36	0.50	1.00	0.31
Sperc	0.30	-0.13	0.21	0.19	-0.04	0.59	0.05	-0.13	-0.62	-0.04	0.31	1.00

Table S3 – Correlation matrix for average coefficients of friction for micro-structured rubber samples (parametrized by diameter/height of micropillars) and for randomly rough plastic samples (parametrized by rms curvature values).

	100/100	100/200	100/300	150/150	150/350	40/120	0.99	1.09	1.76	1.93	3.03	3.29
100/100	1.00	0.59	0.45	0.77	0.58	0.38	0.71	0.77	0.70	0.77	0.72	0.73
100/200	0.59	1.00	0.72	0.69	0.74	0.66	0.48	0.51	0.51	0.52	0.52	0.53
100/300	0.45	0.72	1.00	0.59	0.80	0.78	0.47	0.46	0.45	0.46	0.48	0.50
150/150	0.77	0.69	0.59	1.00	0.71	0.52	0.70	0.78	0.76	0.78	0.78	0.79
150/350	0.58	0.74	0.80	0.71	1.00	0.77	0.46	0.51	0.47	0.49	0.51	0.51
40/120	0.38	0.66	0.78	0.52	0.77	1.00	0.31	0.31	0.35	0.30	0.32	0.33
0.99	0.71	0.48	0.47	0.70	0.46	0.31	1.00	0.95	0.94	0.94	0.96	0.94
1.09	0.77	0.51	0.46	0.78	0.51	0.31	0.95	1.00	0.96	0.96	0.96	0.96
1.76	0.70	0.51	0.45	0.76	0.47	0.35	0.94	0.96	1.00	0.95	0.96	0.96
1.93	0.77	0.52	0.46	0.78	0.49	0.30	0.94	0.96	0.95	1.00	0.96	0.97
3.03	0.72	0.52	0.48	0.78	0.51	0.32	0.96	0.96	0.96	0.96	1.00	0.97
3.29	0.73	0.53	0.50	0.79	0.51	0.33	0.94	0.96	0.96	0.97	0.97	1.00
